# A Novel Dried Blood Spot-LCMS Method for the Quantification of Methotrexate Polyglutamates as a Potential Marker for Methotrexate Use in Children

**DOI:** 10.1371/journal.pone.0089908

**Published:** 2014-02-25

**Authors:** Ahmed F. Hawwa, AbdelQader AlBawab, Madeleine Rooney, Lucy R. Wedderburn, Michael W. Beresford, James C. McElnay

**Affiliations:** 1 Clinical and Practice Research Group, School of Pharmacy, Queen's University Belfast, Belfast, United Kingdom; 2 Aston Pharmacy School, Aston University, Birmingham, United Kingdom; 3 School of Medicine, Queen's University Belfast and Musgrave Park Hospital, Belfast, United Kingdom; 4 Institute of Child Health, University College London, London, United Kingdom; 5 Arthritis Research UK Centre for Adolescent Rheumatology at University College London, University College London Hospital and Great Ormond Street Hospital NHS Foundation Trust, London, United Kingdom; 6 Department of Women's and Children's Health, University of Liverpool, The Alder Hey Children’s NHS Foundation Trust, Liverpool, United Kingdom; Istituto di Ricerche Farmacologiche Mario Negri, Italy

## Abstract

**Objective:**

Development and validation of a selective and sensitive LCMS method for the determination of methotrexate polyglutamates in dried blood spots (DBS).

**Methods:**

DBS samples [spiked or patient samples] were prepared by applying blood to Guthrie cards which was then dried at room temperature. The method utilised 6-mm disks punched from the DBS samples (equivalent to approximately 12 µl of whole blood). The simple treatment procedure was based on protein precipitation using perchloric acid followed by solid phase extraction using MAX cartridges. The extracted sample was chromatographed using a reversed phase system involving an Atlantis T3-C18 column (3 µm, 2.1×150 mm) preceded by Atlantis guard column of matching chemistry. Analytes were subjected to LCMS analysis using positive electrospray ionization.

**Key Results:**

The method was linear over the range 5–400 nmol/L. The limits of detection and quantification were 1.6 and 5 nmol/L for individual polyglutamates and 1.5 and 4.5 nmol/L for total polyglutamates, respectively. The method has been applied successfully to the determination of DBS finger-prick samples from 47 paediatric patients and results confirmed with concentrations measured in matched RBC samples using conventional HPLC-UV technique.

**Conclusions and Clinical Relevance:**

The methodology has a potential for application in a range of clinical studies (e.g. pharmacokinetic evaluations or medication adherence assessment) since it is minimally invasive and easy to perform, potentially allowing parents to take blood samples at home. The feasibility of using DBS sampling can be of major value for future clinical trials or clinical care in paediatric rheumatology.

## Introduction

Juvenile idiopathic arthritis (JIA) and Juvenile dermatomyositis (JDM) are chronic inflammatory disorders that affect children; they have potentially serious consequences such as joint destruction and disability. The aetiology of JIA and JDM suggests the use of anti-inflammatory and immunomodulatory agents to reduce or stop the inflammatory process and achieve disease control [1], [2]. The most important, first line therapy for both these diseases in children and young people is methotrexate (MTX) [3]–[5]. MTX is a folate antagonist that possesses potent anti-inflammatory activity; it can be used alone or in combination with other medications such as corticosteroids leading to control of the inflammation process, and is able to slow disease progression [6]. However, due to the wide spectrum of its side effects and inter-patient variability of clinical response, tolerability and absorption, monitoring of MTX metabolite concentrations is valuable clinically, in clinical trials using MTX and can also be used to assess adherence to prescribed regimens.

There is no clinical value in monitoring serum or plasma concentrations of the drug itself since about 95% of a given dose is metabolised within 24 hours of administration [7]. On the other hand, monitoring methotrexate polyglutamates (MTXPGs), produced in the body from the metabolism of MTX, can act as a biomarker to assess long-term therapy and adherence of MTX in paediatric patients with JIA or JDM. MTXPGs are formed intracellularly through sequential γ-linkage of glutamic acid residues to MTX by the enzyme folylpolyglutamate synthetase (FPGS), resulting in MTXPGs which are retained within red blood cells long after MTX has been eliminated from the serum [8]. Evidence suggests that MTXPGs may also be associated with efficacy and toxicity of the drug in the treatment of adults with rheumatoid arthritis and investigators have advocated their routine monitoring [9]–[11].

Several analytical methods have been described in the literature to quantify methotrexate or individual methotrexate polyglutamates in different human biological matrices. Fluorescence polarisation and enzyme immunoassay methods are available to quantify methotrexate but are focused on the detection of MTX and MTXPGs in plasma and urine and are useful when high dose methotrexate is administered, e.g. in treatment of cancer but are less efficient in quantifying the low concentrations presented when low dose methotrexate is administered, as is the case in JIA and JDM [12]–[14]. Radiochemical-ligand binding assays are sensitive but are expensive and labour intensive [14]. For therapeutic drug monitoring purposes, it has been shown that HPLC methods offer suitable selectivity and sensitivity for the determination of methotrexate and its polyglutamates, particularly when utilising fluorescence detection after post-column photo or electrochemical derivatisation [15]–[18]. Until very recently, however, LC-MS-MS quantification of methotrexate received very little attention. MTXPGs determination was first described by Chen and colleagues who determined MTXPGs in Caco-2 cells [19]. The first method described for the detection of MTXPGs in red blood cells using MS detection was published in 2009, using positive electro-spray ionisation [20].

To date, however, all of the existing methods for measuring MTXPGs levels in patients necessitate blood withdrawal by venipuncture which is invasive, requires relatively large blood samples and needs clinical expertise. The goal of the present study, therefore, was to develop and validate a simple bio-analytical method for measuring MTXPGs in dried blood spots (DBS) which can be applied to clinical practice (e.g. checking adherence to prescribed MTX, absorption difficulties or other pharmacokinetic deviations). The use of dried blood spots overcomes issues around the volume of blood to be taken, is more convenient to transport to the laboratory form remote sites e.g. GP surgeries, and can be used for home sampling where parents or children themselves can provide samples without the need for medical supervision. The present study applies for the first time the novel DBS sampling approach to methotrexate and compares the results obtained with a conventional HPLC methodology for measuring MTXPGs in red blood cells [17].

## Materials and Methods

### Ethics statement

The study was approved by the Office for Research Ethics Committees in Northern Ireland (Ref no. 10/NIR03/33). Patients were included in the study only after their parents or legal guardians had been fully informed and had signed the study consent form. In addition, verbal assent was obtained from older children (≥6 years) before enrolment into the study.

### Reagents and chemicals

MTX was purchased from Sigma-Aldrich (Ayrshire, UK). Methotrexate diglutamate (MTX-PG_2_), triglutamate (MTX-PG_3_), tetraglutamate (MTX-PG_4_) and pentaglutamate (MTX-PG_5_) were purchased from Schircks Laboratories (Jona, Switzerland). Acetonitrile (ACN), ammonia solution (NH_4_OH) and methanol were of HPLC grade and purchased from VWR International (Leicestershire, UK). Potassium hydroxide (KOH) and monobasic potassium phosphate (KH_2_PO_4_) were purchased from VWR International (Leicestershire, UK). Mercaptoethanol and perchloric acid were purchased from Sigma-Aldrich (Ayrshire, UK). Ammonium bicarbonate (NH_4_HCO_3_), formic acid (CH_2_O_2_), hydrogen peroxide (H_2_O_2_) and Hanks' balanced salt solution (HBSS) were purchased from Sigma-Fluka (Ayrshire, UK). Lyophilised human plasma was used as a source of the γ-glutamyl hydrolase enzyme and was purchased from Sigma-Aldrich (Ayrshire, UK). HPLC grade water was produced using a Millipore Direct-Q™ 5 water purification system from Millipore (Watford, England). Guthrie cards (Schleicher & Schuell 903) were purchased from Aston Ltd. (Oldham, England) and Oasis solid phase extraction (SPE) cartridges were obtained from Waters (Dublin, Ireland). All reagents used in this work were of the highest available quality. All mobile phase solutions were filtered under vacuum through FP-Vericel (0.45µm) membrane filters from Sartorius (Epsom, UK) and sonicated for 20 minutes prior to use.

### Preparation of calibration standards

Stock solution (100 µM) of each of MTXPGs was prepared by dissolving the appropriate amount of each compound in 1 ml of 100 mM KOH buffer, and then diluting with water. Working standard solutions of different concentrations of MTXPGs were prepared by appropriate dilutions of the stock solutions with water. These working standards were then used for the preparation of whole blood calibration standards of MTXPGs with final concentrations ranging from 5-400 nM. The whole blood (950µl) was spiked with a calibration mixture of equimolar amounts of all polyglutamates (MTXPG_1–5_) to produce final total MTXPGs concentrations of 400, 300, 200, 100, 62.5, 50, 25, 20 and 10 nM. These concentrations were chosen as they cover the expected MTXPGs concentrations obtained in children receiving MTX therapeutically. Stock solutions were stored at −80°C until required. All working standard solutions were freshly prepared from the stock prior to each analytical run. The QC samples at concentrations of 2.5 (LLOQ), 25 (LQC), 125 (MQC) and 250 nM (HQC) for total MTXPGs and 4 (LLOQ), 10 (LQC), 25 (MQC) and 50 nM for individual polyglutamates were prepared in a similar fashion.

### Sample preparation and extraction

To prepare the blood spots, 30 µL of the spiked blood standards were spotted onto Guthrie cards and allowed to dry overnight in the dark at room temperature. The spotted Guthrie cards were then placed in grease-proof envelopes, covered with aluminium foil and stored in an airtight polypropylene container at −80°C until analysis. RBC lysis occurs during the blood drying process and the freeze-thaw cycle prior to analysis.

#### Determination of total MTXPGs after conversion to MTX

For each DBS, a 6 mm diameter disc was punched manually and placed in a polypropylene Eppendorf tube (2.0 mL capacity) and mixed with 100 µL of water for 1 min. Total MTXPGs were measured after conversion to MTX by adding 50 µL of reconstituted plasma, vortex-mixed for 30 sec, followed by the addition of 100 µL of buffer containing 100 mM KH_2_PO_4_ and 150 mM mercaptoethanol. The mixture was then incubated for 14 hours in the dark at 37°C. After incubation, 750 µL of water was added to the mixture, vortex-mixed for 3 min, and 20 µL of 70% perchloric acid was then added and the whole mixture centrifuged at 10,000 g for 10 min.

The clear supernatant was then subjected to solid phase extraction (SPE) using Oasis MAX cartridges (1 ml/30 mg) on a Waters Extraction Manifold (Waters, USA). The MAX cartridges were conditioned using 1 mL of methanol followed by 1 mL of water. The loaded sample was then washed in three steps firstly, 1 ml of 5% aqueous NH_4_OH was used followed by 1 ml of 100% methanol and then 1 ml of 2% aqueous formic acid. Finally, 0.5 ml of 2% formic acid in water:methanol mixture (60∶40) was used for the elution step. The eluate obtained was evaporated to dryness under a gentle stream of nitrogen at 37°C for 40 min, reconstituted in 100 µL of mobile phase and 45 µL injected for LC–MS analysis.

#### Determination of individual MTXPGs

A 6 mm diameter disc was punched from each DBS and then transferred to a 2.0 ml Eppendorf tube and vortex-mixed with 950 µL of water for 3 min. An aliquot of perchloric acid (20 µL, 70%) was then added and vortex-mixed for 30 sec. The tubes were then centrifuged at 10,000 g for 10 min, and the clear supernatant was then transferred to Oasis MAX cartridge for extraction according to the SPE procedure described above. The eluate was evaporated under nitrogen for 40 minutes at 37°C and then reconstituted in 100 µL of mobile phase. A total volume of 45 µL of the final extract was then injected onto the LC-MS-MS system.

### Chromatographic and mass spectrometric (MS) conditions

Chromatographic separation was achieved using reverse phase chromatography with gradient elution. The chromatographic system consisted of Waters Alliance 2795 Separation Module coupled with a Waters Quattro Premier XE tandem quadrupole mass spectrometer 

(Micromass, Manchester, UK) equipped with an electrospray ionization (ESI) source. The instrument was operated in positive ion mode. An Atlantis T3-C18 column [150×2.1 mm (i.d.); particle size, 3µm; Waters] protected with a guard cartridge [20 mm×2.1 mm; particle size, 3µm; Waters] was used as the stationary phase. The LC system and the mass spectrometer were both controlled by MassLynx 4.0 Software with the QuanLynx Application Manager. The mobile phase consisted of 10 mM NH_4_HCO_3_ buffer adjusted to pH 7.5 using formic acid (A) and acetonitrile (B). Separation of the individual MTXPGs (MTXPG_1–5_) was achieved using a linear gradient from 0% to 20% mobile phase B over 20 min (at a flow rate of 0.15 ml/min). After 20 min, the mobile phase was returned to 100% mobile phase A and re-equilibrated for 10 min. For the analysis of MTXPG_total_, a gradient elution from 8% to 20% mobile phase B over 10 min was utilised (at 0.15 ml/min) followed by 10 min re-equilibration at 98% mobile phase A. For both analyses, the column temperature was maintained at 30°C and the auto-sampler at 4°C.

The different MS parameters including multiple reaction monitoring (MRM) of precursor ions, product ions and their collision energy parameters (Table 1) were optimised by direct infusion of the individual analytes (syringe pump; 10µl/min) dissolved in 80% A and 20% B, closely resembling the chromatographic conditions. During the tuning, the intensity of the base peak for each compound was monitored and adjusted to maximum. A minimum of 2–3 transitions were used for each compound. The transitions which resulted in the most selective and abundant product ions were selected as the quantifier transitions (**Table 1**). MRM mass spectrometer analysis mode was used with the transition (455.4 →175.05) to capture the MTXPG_total_ signal. The following optimised MS operating conditions were used: nebulising gas (nitrogen), 80 l/h; desolvation gas (nitrogen), 800 l/h; source temperature, 135°C; desolvation temperature, 450°C. The dwell time for each ion was set at 0.1 s.

**Table 1:** MRM parameters for the analysis of MTXPGs.

compound	Parent ion (m/z)	Product ion (m/z)	Cone voltage (V)	Collision energy (V)
MTXPG_1_	455.40	175.05	21.00	36.00
MTXPG_2_	584.40	175.05	26.00	50.00
MTXPG_3_	713.40	175.05	38.00	56.00
MTXPG_4_	842.30	175.05	40.00	62.00
MTXPG_5_	971.60	175.05	46.00	67.00

### Assay validation

The following parameters were evaluated for the validation of the developed method [21].

#### Selectivity

The selectivity of the developed method was determined by analysing blank blood spots from different sources (n = 6) and spiked blood spots containing MTX and its polyglutamates at a final concentration that equals the determined lower limit of quantification (LLOQ). Representative chromatograms were generated to show that endogenous components present in the sample matrix were resolved from the analytes of interest using the proposed extraction procedure and chromatographic/mass spectrometric conditions. Potential interference from concomitant medications commonly taken by paediatric patients with JIA or JDM was investigated by analysing samples which had been spiked with the appropriate drugs, e.g. folic acid, prednisolone and omeprazole.

#### Linearity

The linearity of the method was established by constructing calibration curves for both individual MTXPGs (MTXPG_1–5_) and MTXPG_total_, on five consecutive days. Plots of peak area (response) against analyte concentration were used. The slope, the intercept and the correlation coefficient of each calibration curve were determined.

#### Limit of detection and lower limit of quantification

The following equation was used to estimate the limit of detection (LOD) for each compound [21]:
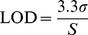
where σ is the standard deviation of the response (estimated from the standard deviation of y-intercepts of regression lines), and S is the slope of the standard curve.

Similarly, the lower limit of quantification (LLOQ) was estimated using the following equation:
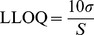



The estimated LOD and LLOQ were subsequently validated by analysis of samples known to be near or prepared at those concentrations.

#### Precision and accuracy

The accuracy and precision of the developed method were determined from the analysis of DBS samples spiked with MTXPGs at four concentrations representing the LLOQ as well as the low, medium and high portions of the standard curves (LQC, MQC and HQC). Intra-day accuracy and precision were calculated on a single day using five replicates at each concentration level. Inter-day bias and precision were calculated using three replicates at each concentration level over five consecutive days. The QC samples were analysed against the calibration curve and the concentrations obtained were compared with the known value.

The accuracy and precision of the method were expressed as the mean percentage relative error (%RE) and percentage coefficient of variation (%CV), respectively. The mean accuracy (%RE) and precision (%CV) should be within 15% of the actual value except for LLOQ which should not deviate by more than 20% [22].

#### Recovery and Matrix effect

The efficiency of the extraction procedure was determined by the analysis of BDS samples spiked with MTXPGs at three concentrations (LQC, MQC and HQC). Five replicates at each concentration level were extracted and analysed and the responses compared with those of non-extracted standards, which represent 100% recovery.

Matrix effect or ion suppression caused by the biological matrix [23] was assessed using the post-column infusion method where an equimolar solution of MTXPs (total concentration of 50 nM) was continuously infused post-column at a rate of 10 µL/min through a T joint and mixed with the column effluent of processed blank DBS samples injected into the column to analyse the potential influence of eluting matrix components on analyte responses. The absolute matrix effect was also measured quantitatively by comparing the ion counts of post-extraction spiked samples with those from pure solutions at three pre-determined concentrations of MTXPGs (LQC, MQC and HQC). If the ratio is <85% or >115%, an exogenous matrix effect is implied [24].

#### Stability

A stability study of MTX and its metabolites over a two month period was conducted to ensure that patient samples are stable during shipping and storage, prior to analysis. The stability of samples during storage was determined by analysing DBS samples containing MTXPGs at high and low QC levels (n = 5 replicates) weekly over two months after storage at −80°C or at room temperature (25°C). For each sample, the ratio of the two concentrations measured for each analyte before and after storage, was calculated. The mean ratio and standard deviation for each analyte was then determined.

#### The effect of the haematocrit on spreadability of blood on Guthrie cards

In order to examine the effect of varying haematocrit (Hct) levels on the spreadability of blood when spotting DBS samples, various Hct levels of whole blood were created by adding plasma to or removing plasma from fresh human blood. Blood was prepared at Hct levels of 30, 40, 45, 50 and 55% and aliquots (30 µL) of the prepared blood samples were then spotted on Guthrie cards and allowed to dry as described previously (n = 8). The spreadability was determined by measuring the surface area of the dried blood spot formed using ImageJ image analysis software.

### Method comparison

In the present study, MTXPG DBS levels measured by the developed LC/MS/MS procedure were compared to those measured in packed RBCs by a conventional HPLC method developed and validated in our laboratory. The developed HPLC method was based on that reported by Dervieux *et al.* [17] because it has been widely used in clinical studies that have served as the basis for proposing treatment-related therapeutic ranges for MTXPGs. The extraction procedure described by Derviuex *et al.* is rapid, easy to perform and allows simultaneous determination of individual MTXPGs or total MTXPGs. However, rapid extraction has led to less than optimal clean-up of the samples which result in the retention of unremoved endogenous components on the guard column and subsequently on the column. Due to these difficulties a combined method involving protein precipitation and SPE clean-up procedure was developed to enhance the removal of endogenous substances from the samples prior to HPLC analysis. Briefly, 50 µL RBC haemolysate were prepared and extracted as detailed by Dervieux et al. but the supernatant was diluted with water up to 1 ml and then subjected to solid phase extraction using Oasis MAX cartridges as described in section 2.3.1. The eluate was evaporated under nitrogen for 40 minutes at 37°C and then reconstituted in 100 µL of mobile phase. A total volume of 50 µL was then injected onto the HPLC system. The chromatographic method employed reverse phase chromatography with post-column photooxidation and fluorometric detection. Separation was achieved using Waters XBridge C18 column (250×4.6 mm ID, 5µm) fitted with a 20 mm guard of similar chemistry, both maintained at 40°C. Emission and excitation wavelengths were similar to those selected by Dervieux et al [17]. Since the HPLC method involved some modifications to that reported by Dervieux *et al.*, it was revalidated in our laboratory according to the ICH guidelines [21]. Results showed that the developed method was linear over the concentration range studied (5–400 nM of MTXPGs) with a correlation coefficient >0.996 for all analytes. Accuracy (% RE) and precision (% CV) values for within and between day were <20% at the LLOQ and <15% at all other concentrations tested. The LLOQ of the method was validated at 4.0 nM for each polyglutamate and 4.5 nM for total MTXPGs.

### Clinical application

The developed assays of MTXPGs in DBS and packed RBCs were applied to the analysis of DBS finger prick samples and whole blood samples collected from children with JIA or JDM who were receiving MTX weekly doses (oral or subcutaneous) for at least two months. Whole blood samples (1 ml) were taken by venepuncture from each patient at the same time the DBS sample was collected. Packed RBCs were obtained from the whole blood sample after a centrifugation step to separate plasma and buffy coat from RBCs, the RBCs were washed twice with two volumes of Hanks' balanced salt solution (HBSS) and packed RBCs were then stored at −80 until analysis. MTXPGs concentrations measured from DBS and packed RBC samples were then compared utilising Bland-Altman plots [25].

### Statistical methods and data analysis

Linearity of the assay was calculated by linear regression analysis. Standard curve regression analysis was performed by MassLynx 4.0 software (Waters Corporation, USA). The level of agreement between the developed LC-MS method and the conventional HPLC method based on that reported by Dervieux *et al.* was examined using Bland-Altman plots generated with GraphPad Prism (ver. 5) software; 95% of MTXPGs levels resulting from the two sampling methods should not exceed ± two times the standard deviation of the mean difference in order to pass the test [25]. Means and standard deviations were calculated using Microsoft Excel 2010 software (Microsoft Corporation, USA).

## Results and Discussion

### LCMS determination of MTXPGs in DBS samples

**Figure 1:**
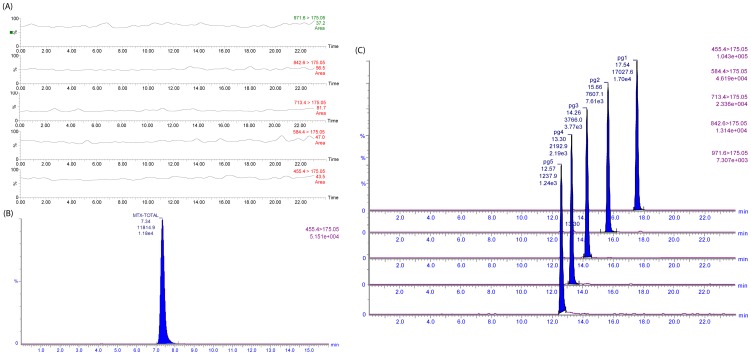
Representative MRM chromatograms of a blank DBS sample (A), DBS spiked with MTXPGs (final total concentration of 100 nM) subjected to enzymatic conversion to MTXPG1 (B), and DBS sample spiked with MTXPG1–5 measured individually at a final concentration of 100 nM each (C).

The developed method was found to be selective for MTX and its metabolites in the presence of endogenous matrix components. Representative MRM chromatograms, obtained following the sample treatment procedure outlined above, demonstrated the resolution of individual MTX polyglutamates (MTXPG_1–5_) and total MTXPGs (after conversion to MTXPG_1_) from the biological matrix of blank DBS samples (Figure 1). The basic pH of the mobile phase (buffered at 7.5) resulted in proportionally higher ionization of the carboxylate groups of longer-chain polyglutamates, resulting in elution inversely proportional to polyglutamation number. The performance of several chromatographic columns (Waters Xbridge C18, Waters Atlantis T3 and Sequant HILIC) was tested for the separation of MTXPGs; however, baseline separation with the highest efficiency and peak symmetry was achieved with Atlantis T3 column. This was particularly true for longer chain polyglutamates as the column provided enhanced retention of such very polar compounds while exhibiting good peak shape and efficiency. The total run time for determining the individual MTXPGs was 20 min with all peaks of interest being eluted within 18 min. However, when total MTXPGs was determined, the total run time was only 10 min with the peak of interest (MTXPG_1_) being eluted within 8 min.

A series of experiments were conducted to ascertain the most appropriate solvent and method for extraction. Following these studies, it was concluded that extraction of DBS with water and protein precipitation using perchloric acid resulted in high and reproducible recoveries. This was followed by The SPE clean up procedure. Strong retention of all MTXPGs was obtained by the use of Oasis MAX SPE cartridges which contain a mixed-mode polymeric sorbent that has been optimized to achieve higher selectivity and sensitivity for extracting acidic compounds with anion-exchange groups. Clean extracts were eluted and evaporated to dryness and reconstituted in a small volume prior to analysis. Positive ESI mode was chosen for the determination of MTXPGs since it produced a more favourable signal to noise ratio, ionized MTXPGs more efficiently and resulted in linear regression curves for the analytes. Fragmentation of MTXPGs using collision induced dissociation at the optimized MS parameters resulted in abundant product ions at *m*/*z* 308.10 and 175.05. The latter product ion was selected to obtain the maximum selectivity for all MTXPGs under the MRM spectra since an unidentified endogenous compound that forms a 308.10 fragment was observed resulting in a peak that could interfere with MTX in the MRM channel.

The selectivity of method was investigated by analysing DBS samples collected from untreated healthy subjects and also from patients with JIA or JDM. No interferences were observed at the retention times of MTPGs. Additionally, no interferences were observed from the drugs commonly given to the study patients (see earlier).

### Method validation

Calibration curves demonstrated a linear relationship between peak area and concentration with correlation coefficients >0.995 for all five individual polyglutamates (MTXPG_1–5_) and for MTXPG_1_ formed after enzymatic conversion of MTXPG_total_. The mean correlation coefficient, slope and intercept values describing the calibration curves are presented in Table 2. Within and between day accuracy and precision were determined for each of the individual MTXPGs and MTXPG_total_ during the 5-day validation experiments at low, middle and high QC concentrations. Precision and accuracy were found to be within ± 15% at all QC concentrations as shown in Table 3. The limits of detection for individual MTXPGs and MTXPG_total_ were determined as 1.6 and 1.5 nM, respectively. The corresponding LLOQ values were calculated as 5 and 4.5 nM, respectively. These values were validated by analysis of a suitable number of samples known to be near the calculated LLOQ (4 and 2.5 nM for individual and total MTXPGs, respectively) [26], [21]. The precision and accuracy at these concentrations were within the acceptable limits of ±20% (Table 3).

**Table 2:** Results of the five calibration curves for MTXPGs; calibration points (nmol/L)^a^, slope±SD, intercept and correlation coefficient of the standard curves.

Compound	C1	C2	C3	C4	C5	C6	Slope (mean±SD)	Intercept (mean±SD)	Correlation coefficient (r)
MTXPG_1_	5 (4.5±0.5)	12.5 (11.3±1.6)	20 (19.4±0.7)	40 (38.7±2.4)	60 (59.9±0.1)	80 (81.0±1.3)	187.8±17.9	−18.9±3.4	>0.998
MTXPG_2_	5 (4.8±0.6)	12.5 (11.4±1.7)	20 (20.5±0.8)	40 (39.3±1.9)	60 (60.0±0.1)	80 (80.4±1.1)	80.1±11.2	49.2±6.1	>0.999
MTXPG_3_	5 (4.8±0.5)	12.5 (11.7±1.2)	20 (20.2±0.5)	40 (39.9±2.5)	60 (59.7±0.2)	80 (80.2±1.4)	46.4±4.2	33.4±3.9	>0.998
MTXPG_4_	5 (4.4±0.7)	12.5 (11.2±1.4)	20 (19.3±1.7)	40 (39.8±1.6)	60 (60.1±0.8)	80 (79.3±0.7)	30.6±4.4	0.52±0.2	>0.999
MTXPG_5_	5 (4.9±0.5)	12.5 (12.0±1.5)	20 (20.4±2.2)	40 (38.8±3.5)	60 (59.9±1.2)	80 (80.6±1.9)	7.6±1.1	17.9±2.7	>0.995
MTXPG_total_	10 (10.9±1.8)	20 (21.8±0.6)	50 (47.8±1.4)	100 (90.5±3.7)	200 (190.9±3.9)	400 (406.9±2.0)	25.6±4.9	−113.9±12.8	>0.998

a Spiked concentrations of calibration standards [measured concentrations (mean±SD) are shown within brackets].

**Table 3:** Results of within day (intra-day) and between days (inter-day) accuracy and precision of MTXPG analysis (n = 5).

Compound	Spiked (nominal) concentration (nmol/L)	Intraday (n = 5)			Interday (n = 5)		
		Measured (mean ± SD) (nmol/L)	RSD^a^, %	Accuracy^b^, RE%	Measured (mean ± SD) (nmol/L)	RSD, %	Accuracy, RE%
**MTXPG_1_**	4 (LLOQ)	4.06±0.19	4.68	1.50	4.12±0.24	5.83	3.00
	10 (Low QC)	9.58±0.84	8.76	−4.20	9.72±0.90	9.25	−2.80
	25 (Middle QC)	25.94±0.98	3.77	3.76	25.54±1.13	4.42	2.16
	50 (High QC)	51.16±1.12	2.19	2.32	49.38±1.78	3.61	−1.24
**MTXPG_2_**	4 (LLOQ)	3.92±0.37	9.44	−2.00	4.22±0.19	4.50	5.50
	10 (Low QC)	9.14±0.93	10.19	−8.60	10.02±1.01	10.08	0.20
	25 (Middle QC)	23.46±1.67	7.11	−6.16	25.92±1.26	4.86	3.68
	50 (High QC)	46.18±3.49	7.55	−7.64	49.86±1.83	3.66	−0.28
**MTXPG_3_**	4 (LLOQ)	4.24±0.52	12.26	6.00	4.14±0.09	2.16	3.50
	10 (Low QC)	10.08±0.95	9.42	0.80	9.78±0.30	3.08	−2.20
	25 (Middle QC)	26.00±0.89	3.42	4.00	25.24±0.40	1.57	0.96
	50 (High QC)	52.00±1.07	2.05	4.00	52.14±3.54	6.79	4.28
**MTXPG_4_**	4 (LLOQ)	4.34±0.17	3.92	8.50	4.14±0.38	9.17	3.50
	10 (Low QC)	10.20±1.19	11.62	2.00	10.28±0.67	6.52	2.80
	25 (Middle QC)	25.10±2.63	10.49	0.40	26.08±1.36	5.21	4.32
	50 (High QC)	51.86±3.63	7.00	3.72	48.74±3.49	7.16	−2.52
**MTXPG_5_**	4 (LLOQ)	4.28±0.83	19.39	7.00	4.52±0.51	11.28	13.00
	10 (Low QC)	11.44±1.09	9.53	14.40	10.62±1.16	10.93	6.20
	25 (Middle QC)	26.06±1.64	6.29	4.24	26.10±0.75	2.87	4.40
	50 (High QC)	49.70±5.75	11.57	−0.60	45.22±1.10	2.43	−9.56
**MTXPG_total_**	2.5 (LLOQ)	2.72±0.39	14.33	8.80	2.99±0.10	3.34	19.60
	25 (Low QC)	25.82±2.87	11.11	3.28	27.85±1.70	6.10	11.40
	125 (Middle QC)	126.66±9.75	7.69	1.32	133.13±5.66	4.25	6.50
	250 (High QC)	246.04±5.26	2.13	−1.58	261.03±18.23	6.98	4.41

a Relative standard deviation (RSD)% = [standard deviation (SD)/mean]×100.

b Relative error (RE)% = [(measured−nominal)/nominal]×100.

**Figure 2:**
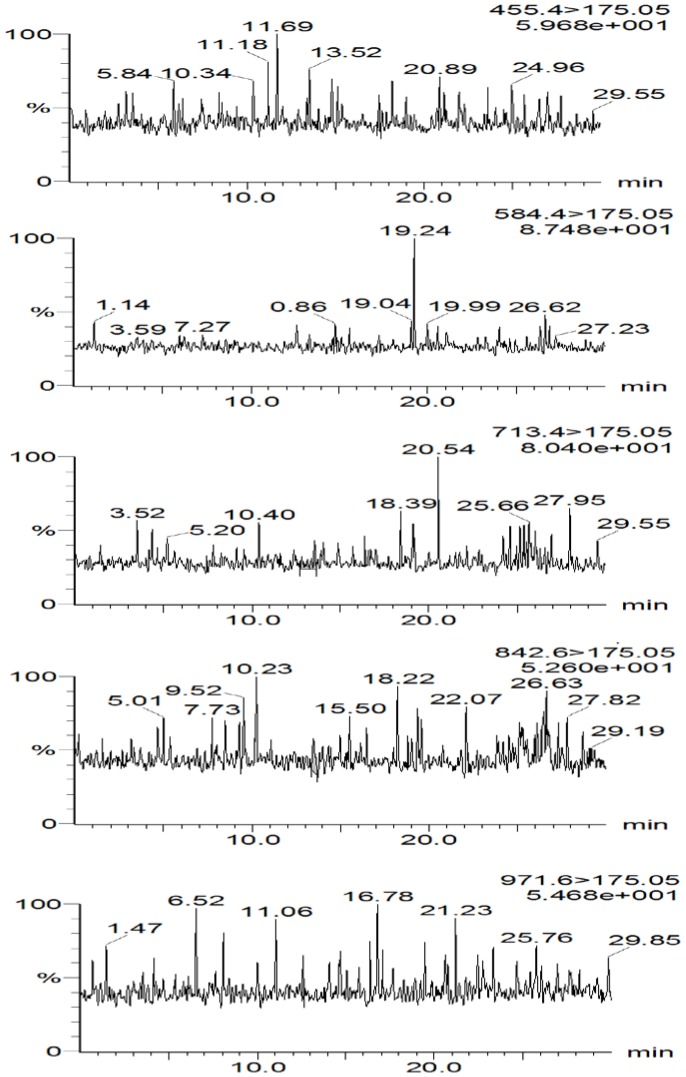
Individual MRM traces obtained upon injection of processed DBS with blank blood into a column post-infused with MTXPG solutions; chromatograms indicated no matrix effect over the time of analysis.

The mean extraction recoveries were 71% for MTXPG_1_, 70% for MTXPG_2_, 72% for MTXPG_3_, 44% for MTXPG_4_, 49% for MTXPG_5_ and 70% for MTXPG_total_. Although lower recoveries were obtained for longer-chain polyglutamates (MTXPG_4–5_), they were reproducible. Such values compared favourably with recently published methods utilising SPE for extracting MTXPGs [20]. Although the extraction of extracellular MTXPGs in spiked standards might not be fully comparable with the extraction of intracellular glutamates in patient samples, any potential difference was minimised in the present study by haemolysing the RBC cells in both standard and patient DBS samples through blood drying and freeze-thawing of samples prior to analysis. The matrix effect, defined as the analyte ionisation suppression or enhancement due to the presence of endogenous components within the biological matrix [24], was assessed using a qualitative method as shown in Figure 2, which present MRM traces obtained during the post-column infusion experiment. The results indicate that there were no significant baseline variations at the retention times of MTXPGs. In addition, a quantitative method revealed that the estimated matrix effect (% ME) on all MTXPGs was within the acceptable limits of ±15%, which indicates the lack of any major ion suppression or enhancement for MTXPGs in this method.

The result of the stability studies indicated that MTXPGs in the DBS matrix were stable at −80°C and room temperature (25°C) over a 2-month storage period. The values found were 0.95±0.06 for MTXPG_1_, 1.09±0.02 for MTXPG_2_, 0.98±0.07 for MTXPG_3_, 1.07±0.07 for MTXPG_4_, 1.01±0.10 for MTXPG_5_ and 0.98±0.06 for MTXPG_total_ at −80°C and 0.94±0.08, 0.90±0.09, 0.96±0.11, 0.88±0.07, 0.86±0.07 and 0.93±0.08 for these compounds at room temperature, respectively, indicating stability of MTXPGs at the storage conditions employed.

**Figure 3:**
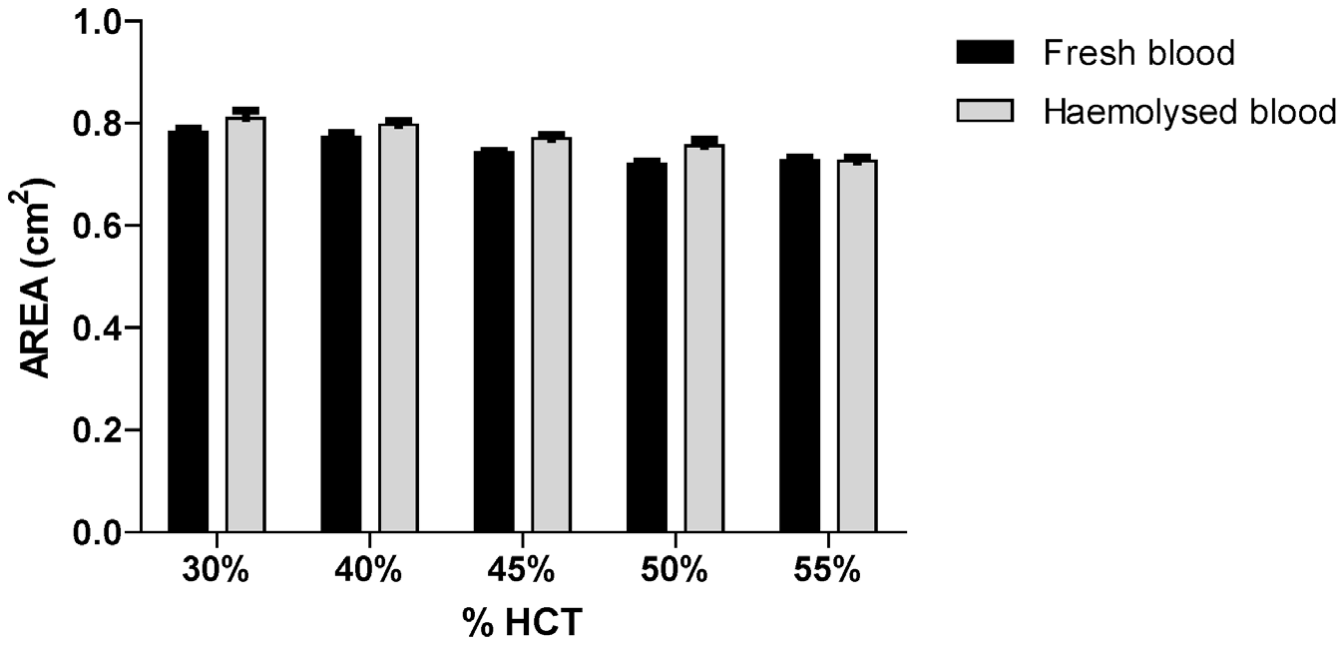
Effect of varying blood haematocrit on the measured area of DBS spot formed (mean area ± SD; n = 8).

Investigation of the distribution of MTXPGs throughout the blood spot applied to the Guthrie card was investigated as described by our group in a previous study [27]. The results indicated that the analytes were evenly distributed throughout the DBS. In addition, the effect of varying Hct levels on the size of the formed spots was assessed. The results demonstrated minimal effect of Hct within the range of 30–55% on the measured surface area of the DBS sample formed when either fresh or haemolysed blood was used (Figure 3). Each of the measured areas displayed a difference of less than ±5% from that measured at the middle Hct level (45%) within the range studied. Haemolysed blood showed a slight increase in blood spot diameter compared to fresh blood with a similar HCT, though the increase in the blood spot size from haemolysed blood was less than 5%.

### Method comparison and analysis of patient samples

The developed LCMS and HPLC assays have been used to determine MTX and its polyglutamates in clinical samples from a cohort of 47 children with JIA/JDM who were receiving low dose MTX treatment (10–20 mg). Matched erythrocyte and DBS samples (n = 94) were obtained in an effort to develop a comparison between the two developed methods. Measured MTXPG levels were within the ranges quoted in literature [28], [17], [20]; MTXPG_3_ was the most predominant species (38.6% of the sum of MTXPG_1–5_) which is consistent with previous publications [17], [20].

**Figure 4:**
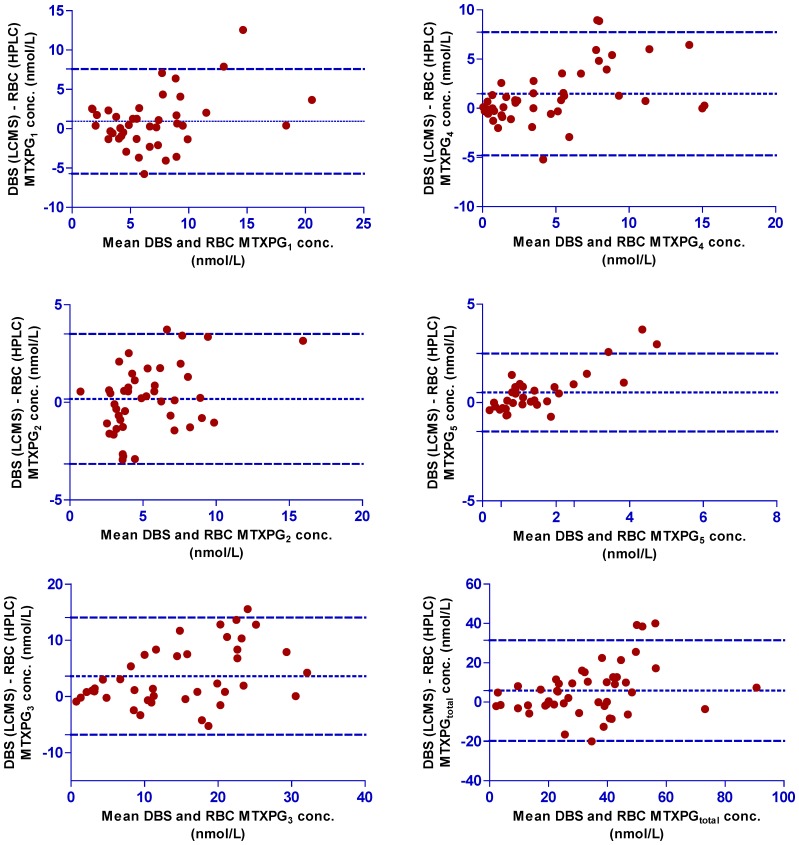
Bland-Altman plots comparing MTXPG_1–5_ and MTXPG_total_ concentrations in DBS versus RBCs in all patient samples investigated (n = 94).

**Figure 5:**
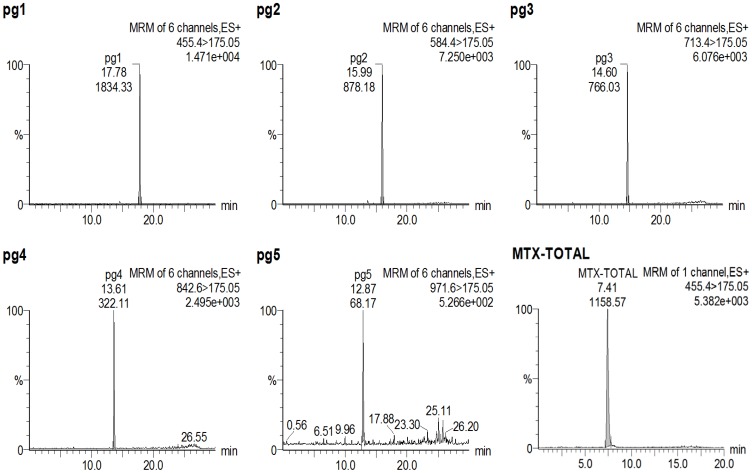
Representative chromatograms of an extracted DBS sample obtained from JIA patient treated with MTX; (pg1–5): MRM chromatograms of individual MTXPGs; concentrations found were MTXPG_1_, 9 nmol/L; MTXPG_2_, 10 nmol/L; MTXP_3_, 15 nmol/L; MTXPG_4_, 10 nmol/L and MTXPG_5_, 7 nmol/L, respectively. (MTX-total): Chromatogram after conversion of total MTXPGs to MTXPG_1_; total concentration found was 49 nmol/L.

To allow comparison with DBS samples, the measured MTXPG concentrations in RBC samples were first converted into the equivalent whole blood concentrations (by taking into account the individual Hct values for each patient). Bland-Altman plots of the difference between DBS and RBC concentrations for each MTXPG were then examined (Figure 4). The mean (*d*) and standard deviation (*s*) of the difference between the two matched readings were calculated to determine the equivalence between the two methods; since more than 95% of the plotted points fell within *d - 2s* and *d + 2s*, the suitability of the DBS analytical method to analyse patient samples was confirmed. Correlation plots between concentrations obtained by the two alternative methods revealed that the difference between the two methods was minimal for short-chain glutamates (MTXPG_1–2_) [as evidenced by a slope of 1.09 (r = 0.81 for MTXPG_1_) and a slope of 1.03 (r = 0.84 for MTXPG_2_)] but increased in magnitude for longer-chain glutamates (MTXPG_3–4_) [slope = 1.22, r = 0.83 for MTXPG_3_ and slope = 1.29, r = 0.84 for MTXPG_4_] with measured concentrations being slightly higher in DBS samples. This suggests that longer-chain glutamates were more easily extracted from DBS compared to erythrocyte samples. We speculate that this is due to enhanced lyses of RBCs when dried on Guthrie cards. Dervieux *et al.* have stated that a higher number of glutamic residues might affect the stability, and therefore, the recovery of MTXPG moieties during the deproteinization step of the conventional HPLC method. This is of particular clinical interest since longer-chain MTXPGs are associated with increased potency over short-chain glutamates. A typical patient chromatogram from an extracted DBS sample is shown in **Figure 5** **.**

## Conclusion

The development and validation of LCMS method designed for the determination of MTXPGs in DBS samples has been described. To our knowledge, this is the first report describing the simultaneous determination of MTX and its polyglutamates in dried blood spots. This novel sampling technique coupled with LC-tandem MS detection system led to a selective and sensitive method appropriate for use in very low volume paediatric samples (approximately 12µl of whole blood). The developed method was shown to be linear, accurate, precise and reliable. The sample treatment procedure is simple, involving protein precipitation followed by SPE clean-up and analyte reconstitution. The analytical method shown here has been successfully applied for the analysis of DBS samples obtained from finger pricks in paediatric patients with JIA and JDM. The methodology has a potential for application in a range of clinical studies (e.g. pharmacokinetic evaluations or medication adherence assessment) since it is minimally invasive and easy to perform, potentially allowing parents to take blood samples at home. The feasibility of using DBS sampling to measure MTX in children, therefore, can be of major value for future clinical trials or clinical care in paediatric rheumatology patients and indeed further studies, particularly relating to methotrexate absorption and medication adherence are at an advanced stage of development by the group.
